# Design and Quantitative Analysis of Cancer Detection System Based on Fluorescence Immune Analysis

**DOI:** 10.1155/2019/1672940

**Published:** 2019-12-24

**Authors:** Lei Shao, Longyu Zhang, Shilin Li, Pengyuan Zhang

**Affiliations:** ^1^Tianjin Key Laboratory for Control Theory & Applications in Complicated Systems, Tianjin University of Technology, Tianjin 300384, China; ^2^Tianjin Xinuo Biomedicine Co., Ltd., Tianjin, China; ^3^Tianjin Yirui Biotechnology Co., Ltd., Tianjin, China

## Abstract

Human blood is an important medical detection index. With the development in clinical medical detection instruments and detection technology, the requirements for detection accuracy and efficiency have been gradually improved. Fluorescent immunochromatography is a new detection technique. It has the characteristics of high efficiency, convenience, no pollution, and wide detection range. Human blood can be detected quickly using fluorescent immunochromatography. At present, it has received great attention from the field of clinical testing. In this paper, a set of fluorescent immunochromatographic analyzer has been designed. It is mainly based on the principle of fluorescence immunochromatography. A new method of signal analysis and system design for fluorescent immunochromatography analyzer is proposed. By using the improved threshold function denoising algorithm, the quantitative detection of fluorescent immunochromatographic strip is realized. The concentration of pathogenic factors (cancer cells) in human serum can be measured conveniently and accurately. The system integrates many peripheral modules, including fluorescence signal acquisition, fluorescence signal processing, quantitative curve fitting, and test results. In this paper, the quantitative detection experiments of the system are carried out in three aspects: linearity, repeatability, and sensitivity. The experimental results show that the linear correlation coefficient is up to 0.9976, and the limit of detection is up to 0.05 ng/ml. The requirements of the system are satisfied. The system performance is good, and the quantitative result is accurate. Therefore, the establishment of a fluorescence analysis system is of great significance.

## 1. Introduction

With the improvement in medical standards and technology, testing instruments are constantly improving. It is developing in the direction of simple operation, accurate results, and higher detection efficiency. At present, immunology is the conventional method for clinically testing blood and body fluids, which is used for quantitative or qualitative analysis [1, 2]. Quantitative immunoassay is an immunological method for quantitative detection of various physiological and pathological indexes in samples based on the principle of antigen-antibody reaction or supplemented by various marker-tracer techniques and special detection equipment. It has the characteristics of high sensitivity and high specificity. Antigens and antibodies are specific and sensitive, so fluorescent immunochromatography has been widely used in the detection of trace substances in clinical specimens [3, 4]. The most common methods are the radioimmunoassays, the enzyme-labeled immunoassay, the chemiluminescence immunoassay, and the colloidal gold immunoassay. These analytical techniques have played an important role in biology, medicine, and other fields since they came out one after another in the middle of the 20th century. In recent years, with the automation of analytical methods and the commercialization of matching reagents, quantitative immunoassay technology has been more and more widely used in clinical laboratories and has become an important means of disease diagnosis and efficacy evaluation. In general, the detection of blood in vivo by medical workers is the colloidal gold immunoassay, and its marker is colloidal gold, which is combined with the labeled antibody protein by physical adsorption, and then precipitates and produces color [5]. The most obvious characteristics of this method are convenient operation, low cost, and high stability, so it is very suitable for hospital and family use. Colloidal gold immunochromatography has the following advantages: observe the results directly with the naked eye without any instrument and equipment, rapid detection, good stability, and it has no toxicity to the operator and no pollution to the environment. However, these characteristics are mainly reflected in the qualitative detection of solid-phase immunity, which cannot meet the clinical needs of accurate and quantitative analysis. It uses human eyes to identify, especially in the case of weak positive, which is easy to lead to missed detection, so the colloidal gold immunoassay is more suitable for semiquantitative and manual qualitative detection, but it is difficult to meet the requirements of quantitative detection. At the same time, the external noise will cause error to the detection results of the colloidal gold immune method, and its markers are only gold markers, and other markers cannot be used.

In recent years, with the development in fluorescence labeling technology, the combination of fluorescence labeling technology, immunochromatographic reaction, and photoelectron analysis technology, a detection technology called fluorescence immunochromatography is widely used in the field of medical detection [6]. It is mainly used in the diagnosis of bacteria, viruses, and serum antibodies. According to the characteristics of color and photoluminescence, the researchers combined it as a fluorescent marker with the substance to be tested, successfully applied it for the detection of immunochromatographic strip, and realized the detection of sample concentration. Compared with the colloidal gold immunochromatographic method, the fluorescence immunochromatographic analyzer has the advantages of convenient testing and simple operation and overcomes the two major defects of low sensitivity and unstable detection results. The fluorescence immunochromatographic detection method has the characteristics of strong specificity, high sensitivity, and good repeatability. At present, it has become a future development trend in the fields of biomedicine and other related fields.

Therefore, a set of fluorescent immunochromatography analyzer based on fluorescence immunochromatography is designed to improve the sensitivity of traditional methods. According to the principle of fluorescence immunoassay, the appropriate fluorescein was selected, and the best wavelength was selected according to its characteristic spectral characteristics to select the excitation light source. Based on the basic principle and system requirements of fluorescent immunochromatography, the design scheme of the system is determined. At the same time, because of the noisy characteristics of fluorescence signals, the wavelet transform method is introduced into the quantitative detection of fluorescent immunochromatography analyzer. Due to the deficiency in traditional wavelet threshold denoising, this paper proposes an improved threshold function method and verifies its feasibility. After a large number of repeated experiments, the test results show that the design system can basically achieve the desired results and can achieve patient management information and more accurate measurement of solution concentration. If cancer patients can seek medical treatment in time, the design instrument can be used to predict the pathological changes, so as to meet the needs of hospitals and patients.

## 2. Fluorescence Processing

### 2.1. Principle of Immune Chromatography

**Figure 1:**
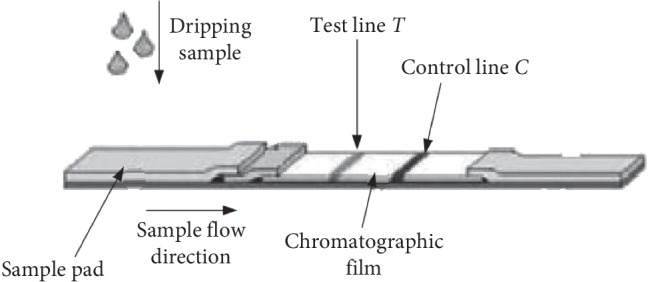
Schematic diagram of immunochromatography.

Immunochromatography is also called immunoaffinity chromatography. The specific antigen-antibody binding reaction has high affinity and specificity. Antigen or antibody can be coupled to column packing by using immunochromatography to prepare an affinity chromatography column. The specific immune components that are simple with antigens or antibodies can be quickly and efficiently isolated and purified from complex mixed samples by the chromatography column. It is the most selective and effective method for isolation and purification of specific antibodies [6, 7]. The immunochromatographic reaction was carried out on the nitrocellulose membrane of the reagent strip. The main immunochromatographic strip has the characteristics of strong maneuverability, high accuracy, and no pollution, which is mainly composed of sample pad, binding pad, nitrocellulose membrane, and water absorption filter paper [8]. Immunochromatographic reaction is on the cellulose membrane and the control zone. The realization of chromatography requires a mobile phase and a stationary phase [9, 10]. The mobile phase is a substance that flows transversely in the reagent strip, and the stationary phase is a nitrocellulose membrane on the reagent strip, which mainly exists in the test zone and the control zone. When the mixture flows through the stationary phase with the mobile phase, its different substances are separated by affinity chromatography [11]. *T*-line (test line) has specific immune response, and *C*-line (control line) has specific immune response, as can be seen in Figure 1.

**Figure 2:**
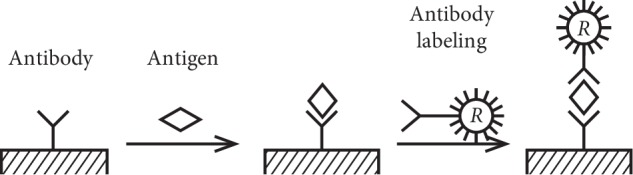
Immune schematic diagram of the double-antibody sandwich method.

In this paper, double-antibody sandwich method was used to detect the concentration of samples. It is carried out on the basis of the indirect method of fluorescent immune reaction. The fluorescein used in the immunochromatographic strip is a europium antibody chelate. It forms a europium-resistant compound by adding an enhancer containing *β*-diketone ligand. A schematic diagram of the principle of immune response by double-antibody sandwich method is showed in Figure 2.

The specific processes are as follows: a specific antibody containing the marker is dropped on the binding pad, and a specific antibody is added at a location of the nitrocellulose membrane. After the sample liquid of the sample to be tested is added to the sample pad, through the action of the capillary tube, the sample solution and the marker are uniformly diffused at the binding pad. The sample solution reacts with the europium chelate to form the complex of the europium antibody chelate. The complex containing the europium antibody chelate then flows forward. When the complex moves to the detection band, because the detection band contains a specific antibody, it has a specific immune reaction with the europium antibody chelate-containing antigen and finally forms the immune complex of antigen-antibody-europium antibody complex. At the same time, the labeled antibody was also captured on the test line and stayed in the test line, while the redundant markers continued to flow forward and were adsorbed to the control band by the protein antibody on the membrane, and the complex specific binding occurred on the control line, and at the same time, the labeled antibody was also captured on the control line. According to the difference of marker types, the signal is detected by color or instrument, so as to achieve the purpose of qualitative or quantitative detection.

### 2.2. Traditional Wavelet Denoising Function

In the process of quantitative detection by using a fluorescence immunochromatography analyzer, the collected fluorescence signal will be affected by external or internal noise, which will lead to inaccurate quantitative detection results. External noise is caused by external environment and human factors. Internal noise includes thermal noise, particle noise, low-frequency noise, and so on. Common filtering algorithms include sliding average filter and wavelet analysis [12]. Wavelet analysis is a new branch of mathematics, which is based on the development of Fourier transform [13–15]. The main characteristics of wavelet analysis are that the time is subdivided at high frequency, and the frequency is subdivided at low frequency, which satisfies the analysis of the time-frequency signal according to different conditions. Therefore, wavelet analysis is a new localization analysis in time and frequency domains, but it is more beneficial to deal with nonstationary signals than Fourier transform. This method not only preserves the ability of local analysis of Fourier transform but also adapts to the window and shape of wavelet according to the characteristics of different signals [16]. There are three kinds of common wavelet analysis: wavelet transform modulus maxima method, spatial correlation filtering denoising, and wavelet threshold denoising. The principle of the wavelet threshold denoising method is simple. The wavelet decomposition coefficients of the original and noise signals are different and processed. And the calculation is relatively small, and the noise can be eliminated almost completely. In this paper, according to the characteristics of white noise in the collected fluorescent signal, the wavelet threshold denoising method is selected to improve the accuracy of the fluorescent signal.

After wavelet decomposition, the energy of the signal is mainly concentrated in some large wavelet coefficients, and most of the noise is the wavelet coefficient with small amplitude [17–19]. Assuming that the original signal is a (*t*), the contaminated noise signal is *b* (*t*) and the noise signal is *n* (*t*), and then the noise-containing basic model may be expressed as represented in the following equation:

(1) $$\begin{array}{c} b\left(t\right)=a\left(t\right)+n\left(t\right). \end{array}$$

Formula (1) is subjected to wavelet transform to obtain the following equation:

(2) $$\begin{array}{c} b_{j,k}=a_{j,k}+n_{j,k}. \end{array}$$

As shown in formula (2), *b*_*j*,*k*_ represents the wavelet coefficients containing the signal *b* (*t*), and *a*_*j*,*k*_ and *n*_*j*,*k*_ represent the wavelet coefficients of the original signal *a* (*t*) and the noise signal *n* (*t*), respectively.

**Figure 3:**
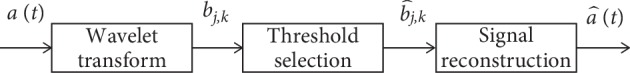
Basic principle block diagram of wavelet threshold denoising.

The amplitude of the wavelet coefficient of the original signal is larger than that of the noise signal, and most of them are distributed in the low-frequency wavelet coefficient. Therefore, a suitable threshold *T* is selected, and the wavelet coefficient threshold is processed to obtain the wavelet coefficient ${\hat{b}}_{j,k}$ after threshold quantification at different scales, and ${\hat{b}}_{j,k}$ is reconstructed to obtain the denoised signal $\hat{a}\left(t\right)$. The specific process is shown in Figure 3.

### 2.3. Threshold Selection and Threshold Function Selection

Multiresolution thoughts are the basis of wavelet transform. The combination of the wavelet transform and the threshold method can effectively remove the noise in the signal [20]. The threshold selection of wavelet denoising will affect the effect of denoising. If the threshold of selection is too large, the useful signal may be eliminated as a signal in the process of denoising, resulting in signal distortion. If the selected threshold is too small, the noise cannot be completely eliminated, which will affect the experimental results. The commonly used threshold selection is as follows: VisuShrink, Rigrsure, Sqtwolog, Heursure, and Minimax. In this paper, the global unified VisuShrink threshold is selected for denoising. VisuShrink can be seen as a universal threshold selector. It provides near-optimal error properties. It also ensures that estimates are as stable as true basic functions. It uses a threshold value *T*_1_, which is proportional to the standard deviation of the noise. It is defined as follows:

(3) $$\begin{array}{c} T_{1}=\sigma_{n}\sqrt{2\;\ln\;N}. \end{array}$$

In formula 3, *σ*_*n*_ is the estimate of noise deviation present in the signal and *N* represents the signal size or number of samples.

Wavelet threshold denoising is to decompose the signal into *L*-layer discrete binary wavelet as wavelet coefficients. Each layer has a fixed threshold. Processing of high-frequency coefficients is decomposed by wavelet using different threshold functions in the wavelet threshold denoising algorithm. It is also important to select a suitable threshold function. In general, there are two kinds of wavelet threshold functions: hard threshold function and soft threshold function.

If the setting *T*_1_ is the threshold, the hard threshold function may be expressed as follows:

(4) $$\begin{array}{c} S=\left\{\begin{array}{c} X,\left|X\right|>T_{1},\\ 0,\left|X\right|\leq T_{1}. \end{array}\right. \end{array}$$

The hard threshold function decomposes the noisy signal wavelet and obtains the high frequency coefficient, which preserves the wavelet coefficient whose absolute value is greater than the threshold value. When the wavelet coefficient is less than the threshold, the wavelet coefficient is set to zero. Hard threshold can protect local characteristics. Therefore, the wavelet coefficients of the whole wavelet domain will have a certain influence on the accuracy of the denoising result in the whole wavelet domain due to the discontinuity in the reconstructed signal.

If *T*_1_ is set as the threshold, then the soft threshold function can be represented as shown in the following equation:

(5) $$\begin{array}{c} S=\left\{\begin{array}{c} \text{SIGN}\left(X\right)\left(\left|X\right|-T_{1}\right),\;\left|X\right|>T_{1},\\ 0,\left|X\right|\leq T_{1}. \end{array}\right. \end{array}$$

The soft threshold function sets the wavelet coefficient greater than its threshold value as the difference between the wavelet coefficient and the threshold value. And the wavelet coefficients less than the threshold are set to zero. The result of the process may be relatively smooth, and there will be a blurry at some of the contours or edge locations.

### 2.4. The Improved Threshold Function

The wavelet coefficients after denoising by the hard threshold method are discontinuity although they can better retain the effective part of the original signal. However, the reconstructed signal after denoising will get the unsmooth signal curve, which will lead to the concussion of the reconstructed signal. The problem that the denoising effect of hard threshold function is not obvious can be solved by using soft threshold function denoising, but the soft threshold function generates a fixed difference in the processing process, resulting in reduced signal accuracy after the reconstruction and also distortion of the reconstructed signal amplitude. In the practical application, it is necessary to carry out the derivation operation on the soft threshold function, but the derivative of the soft threshold function is not continuous and has certain limitations.

In order to take into account the advantages of these two thresholds and eliminate their disadvantages, this paper proposes a new function to solve the soft and hard threshold functions based on the shortcomings of the above threshold functions, as shown in the following equation:

(6) $$\begin{array}{c} {\hat{a}}_{j,k}=\left\{\begin{array}{c} \text{sign}\left(a_{j,k}\right)\left(\left|a_{j,k}\right|-\frac{T_{j}}{1+\log_{2}^{\left(1+\left|a_{j,k}\right|-T_{j}\right)}}\right),\;\left|a_{j,k}\right|\geq T_{j},\\ 0,\;\left|a_{j,k}\right|<T_{j}, \end{array}\right. \end{array}$$

where *a*_*j*,*k*_ is the original wavelet coefficient, ${\hat{a}}_{j,k}$ is the wavelet coefficient after threshold processing, and *T*_*j*_ is the threshold. Through mathematical analysis, the continuity, progressiveness, and deviation of threshold function in formula (4) are verified, and the feasibility of threshold function is proved.

As |*a*_*j*,*k*_| increases, the improved threshold function eventually approaches *a*_*j*,*k*_. The deviation between the reconstructed wavelet coefficient and the actual wavelet coefficient is also gradually reduced. The corresponding denominator also increases. It leads ${\hat{a}}_{j,k}$ to *a*_*j*,*k*_. Thus, the defects of soft and hard thresholds are overcome, and the problem of deviation in ${\hat{a}}_{j,k}$ and *a*_*j*,*k*_ is solved. Because the wavelet coefficient of noise decreases with the increase in the decomposition scale, the threshold in different decomposition layers should decrease with the increase in the decomposition scale. At the same time, in order to solve the common threshold defect, an improved threshold selection method is proposed in this paper. The threshold selected in this article is as shown in the following equation:

(7) $$\begin{array}{c} T_{nj}=\sigma_{j}\frac{\sqrt{2\;\ln\;N_{j}}}{\ln\left(e+j\right)}, \end{array}$$

where *σ*_*j*_ is the standard deviation of layer *j* noise signal, *N*_*j*_ is the length of signal, and *T*_*nj*_ is the threshold of layer *j*.

**Figure 4:**
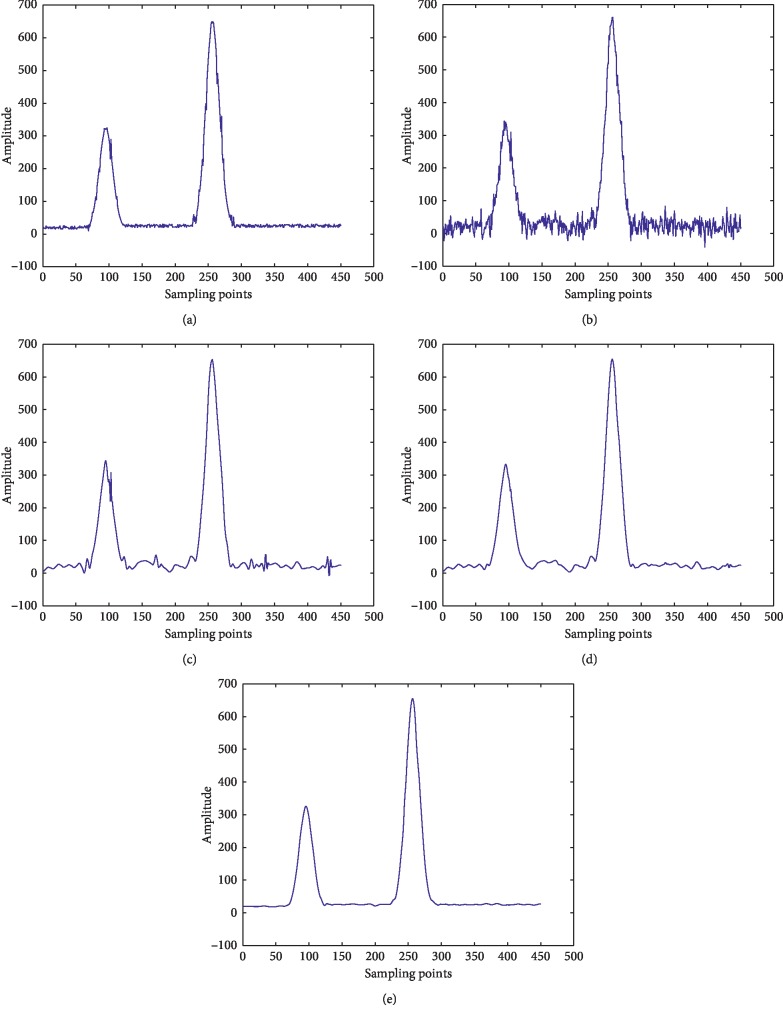
Different kinds of threshold function denoising images: (a) original signal; (b) noisy signal; (c) signal after hard threshold function denoising; (d) signal after soft threshold function denoising; (e) signal of a new threshold function denoising.

The new threshold function is more effective than the traditional threshold function to eliminate the oscillation of the reconstructed signal, and the noise reduction effect is better. The details are presented in Figure 4, where (a) is the original signal, (b) is the noisy signal, (c) is the signal after hard threshold function denoising, (d) is the signal after soft threshold function denoising, and (e) is the signal of a new threshold function denoising.

Signal evaluation standard is the criterion to judge the effect of signal processing [21]. The commonly used indexes to evaluate the quality of the signal are signal-to-noise ratio (SNR) and root mean squared error (RMSE). SNR is defined as follows: the ratio between the energy of the original signal and the noise, where *x* (*n*) is for the original signal, *y* (*n*) is for the noise signal, and *N* is the signal length. The formula is shown as follows:

(8) $$\begin{array}{c} \text{SNR}=10\;*\;\mathrm{lg}\;\left(\frac{\overset{N}{\underset{n=1}{\sum }}x{\left(n\right)}^{2}}{\overset{N}{\underset{n=1}{\sum }}{\left[x\left(n\right)-y\left(n\right)\right]}^{2}}\right). \end{array}$$

RMSE is used to judge the error between the original signal and the noise signal. The formula is shown as follows:

(9) $$\begin{array}{c} \text{RMSE}=\sqrt{\frac{\overset{N}{\underset{n=1}{\sum }}{\left[x\left(n\right)-y\left(n\right)\right]}^{2}}{N}}. \end{array}$$

According to the definition, the smaller the mean square error is, the better the denoising effect is. As shown in Table 1, it compares the SNR and RMSE of the three denoising methods.

In comparison of the denoising index, the improved threshold function denoising method is used to make the fluorescent signal have a higher signal-to-noise ratio (SNR). Root mean square error is low. The effect is better.

## 3. System Design

### 3.1. System Selection and Structure

There are two options for the system. The first option is based on the rapid development of digital image processing technology and the use of a CCD camera to take a photograph of a test strip throughout the detection area [22, 23]. A digital image processing algorithm is used to analyze and calculate the gray value of the analyte concentration and the wavelength band of the detection area and the control area. The second option can eliminate the defect of capturing the image data during the mechanical transmission process and shorten the time. The second option is adding a mechanical scan structure to a legacy optical module. The light is excited by the optical module, and the optical path is concentrated on the test strip. The fluorescence is excited on the test strip and focused on the photodetector. The optical module uses the preamplifier circuit to complete the photocurrent acquisition, and the optical module can complete the fluorescence signal acquisition on the test rod. The mechanical transmission can drive the optical module or place the test rod platform in the direction, which can form a straight line in these two ways. It can complete the entire detection area of the fluorescence collection. The fluorescence curve of the entire detection area is scanned, and then the fluorescence curve is processed to infer the contents of the analyte. This scheme is mature and of low cost, the light module is very small, and this is a common product scheme [24] in the market. The fluorescent immunochromatography analyzer designed in this paper is mainly used in hospitals, families, and other places. Therefore, the system is mainly considered in person from low power consumption, strong anti-interference ability of signal acquisition, and so on. Compared with the above two solutions, we choose the second solution from the perspective of current technological maturity.

**Figure 5:**
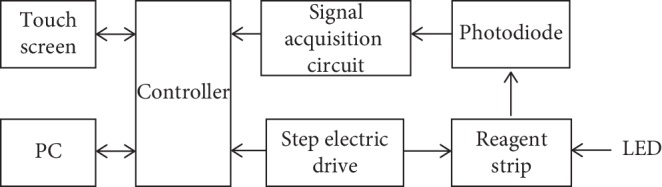
Structure of a fluorescence immunoassay system.

The hardware system is mainly composed of optical system and control system. The main contents of the work are as follows: the driving scanning reagent strip of the motor amplifies the adopted signal and converts the optical signal into electrical signal under the action of fluorescence immunochromatography reaction and realizes the signal acquisition. The software system is mainly composed of touch screen and PC. It can complete the analysis of the signal and carry out data storage and query. The system framework of the fluorescence immunochromatographic analyzer is shown in Figure 5.

The specific work flow of the detection system of fluorescent immunochromatography analyzer designed in this paper is as follows. First, the excitation light generated by the internal timer of the single-chip microcomputer passes through the filter, and the whole reagent strip is scanned under the motion of the drive driven step motor. Silicon photodiode receives the optical signal and converts optical signals into electrical signals. Then, the data are transmitted to the single-chip computer through the *A*/*D* sampling. Finally, the obtained data are sent to the single-chip microcomputer through the serial port, and the collected signals are processed by the single-chip microcomputer. After calculating the concentration value, the detection results are displayed and outputted to the user.

### 3.2. Selection of Light Source

In the fluorescent immunochromatographic reaction, different markers should be selected to correspond to different light sources [25, 26]. In the traditional optical system, the optional types of light sources are xenon lamp, laser, and LED lamp. The advantages of xenon lamp light source emit a wide range of spectra which is from infrared to ultraviolet. The emission intensity of the xenon lamp is very high. The disadvantage of the xenon lamp light source is that the light effect is low, it needs to be under high voltage, and the requirement of power supply is very high. The laser light source has the advantages of a small output pulse width, high signal-to-noise ratio, and high sensitivity. But it has short service life, small number of pulses per second, and large volume, which is not conducive to the portability of the instrument. Although the LED light source is poor in monochromaticity and brightness, it has the advantages of small volume, output power, stable beam wavelength, and long lifetime. Combined with the needs of this system, LED light source is more suitable as the excitation light source of fluorescent immunochromatography analyzer.

The function of the filter is to filter out the interference of radiation from other spectral segments of the system. In the receiving optical path, the bandpass filter needs to be selected. And the peak of the transmission peak is 610 nm. Therefore, a filter having a center wavelength of 610 nm, a peak transmittance of *T*_*s*_ > 90%, and a cut-off depth *T*_*p*_ of less than 0.1% is selected.

## 4. Quantitative Detection Experiments and Results

### 4.1. Linearity Test

The sample solution of *Cryptococcus* pods-like polysaccharide with the concentration of 100 ng/ml was divided into several parts. The solution was diluted to 0.05 ng/ml, 0.1 ng/ml, 1 ng/ml, 10 ng/ml, and 100 ng/m with evaporated feed water. The values of the control line and the test line are measured, respectively, and the characteristic values are obtained, as shown in Table 2.

The fluorescence intensity of the control band and the detection band on the test strip reflects the degree of reaction of the antigen and the antibody. The characteristic value in Table 2 is the ratio of the fluorescence intensity of the control band to the detection band, which indicates the number of fluorescent immune complexes.

**Figure 6:**
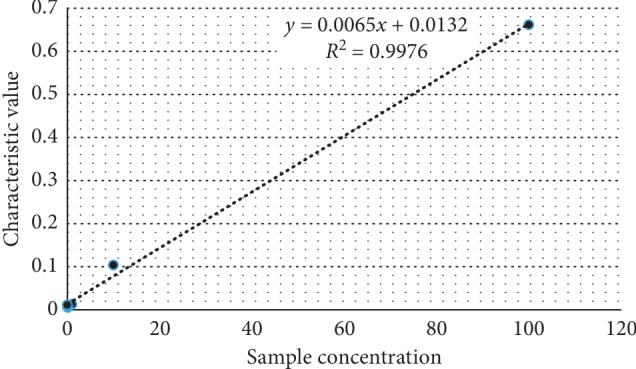
Fitting curve of the linearity test.

The linear correlation coefficient of the fluorescent immunoanalyzer used in this paper is *R*^2^ = 0.9976, as shown in Figure 6. It is shown that the detection system improved by this instrument has the best linear characteristics and can truly reflect the concentration of the object to be measured.

### 4.2. Repeatability Test

The repeatability of the detection results is an important index to test the performance of the fluorescent immunochromatography analyzer. According to the three concentration ranges of low, medium, and high, the experiment was prepared into three concentrations of 1 ng/ml, 10 ng/ml, and 100 ng/ml. In the same location, the same operator uses the same device to repeat the operation 3 times. The experimental data are shown in Table 3.

The coefficient of variation (CV) can be obtained by using the following equation:

(10) $$\begin{array}{c} \text{CV}=\frac{\bar{X}}{\sigma }\;*\;100\%, \end{array}$$

where *σ* is the standard deviation of the sample and $\bar{X}$ is the average value of the sample.

After statistics, the instrument was used to test the sample solution with a concentration of 1 ng/ml, 10 ng/ml, and 100 ng/ml using an improved algorithm. The measurement results show that the repeatability of the instrument at high concentration can be better. In medium and low concentrations, the repeatability of low concentrations is relatively poor. But overall, it shows that the detection system has good repeatability and meets the requirements of the system.

### 4.3. Minimum Test

Take the same batch of the reagent strip. The sample concentration was 100 ng/ml, and the release concentration was 100 ng/ml, 10 ng/ml, 1 ng/ml, 0.1 ng/ml, and 0.05 ng/ml. The solution was dripped on 5 strips and blank strips, respectively, to test it as a detection limit. The specific test data are given in Table 4.

As shown in the table, when the sample concentration is 0.05 ng/ml, the characteristic values are close to those of the blank test paper. Therefore, the limit of *Cryptococcus* capsule polysaccharide solution can be measured by this system.

## 5. Discussion and Conclusion

The system of fluorescent immunochromatography analyzer mainly adopts optical measurement and control module. The invention can realize the quantitative detection of the fluorescent material on the reagent strip under the detection of the concentration of the fluorescent substance solution. The measurement results are reliable and accurate. Through the experiment, we get the following data: the linear correlation coefficient is up to 0.9976, and the limit of detection is up to 0.05 ng/ml. These data prove that the system has the advantages of convenient operation, low power consumption, good stability, and high precision.

The fluorescent immunochromatography analyzer designed in this paper has basically completed the core function. However, due to limited time and lack of time to carry out and explore in depth, there is still a need for improvement in many areas:

1. In the process of sample detection, it will bring noise to the outside and inner boundaries of the system. The next step is to improve the optical system and build more sophisticated optical system modules to stop the noise. At the same time, an amplification circuit with adjustable gain is selected in the control system. The range of fluorescence signals can be controlled within a certain range. And the detection accuracy can be improved.
2. The degree of automation of the system needs to be improved. In this paper, the signal denoising and eigenvalue solution are based on Matlab software. The next plan can be completed in the upper computer system instead of using Matlab to complete the data processing.
3. The fluorescence immunochromatographic analyzer is still being developed and tested. It is not officially in use. A lot of data are needed before formal investment. And we need a lot of samples.

At present, most of the fluorescence signal curves measured by the fluorescence immunochromatographic analyzer are of great noise. They fit most of the curves in the way they deal with noise, and this will bring errors to the system analysis for quantitative analysis. And most of them only stay in theoretical research, and the experimental results are less. Therefore, in this paper, in view of the problems existing in the development, the in-depth study is carried out, and a large number of existing problems are analyzed and solved. At present, there are also many medical enterprises into the research and development of fluorescent immunochromatography analyzer. Although the performance of the instrument has been improved, the cost and maintenance costs are too high, despite the fact that a number of enterprises and research institutes have been well-studied. As a result, the level of technology is to be improved, and further improvements in product innovation and technology upgrading are needed.

## Figures and Tables

**Table 1:** Evaluated parameters for test signals.

Signal	RMSE	SNR
The hard threshold function	11.8552	20.6510
The soft threshold function	10.3338	21.8439
The improved threshold function	7.1187	25.0811

**Table 2:** Linearity test.

Sample concentration	The value of *T*	The value of *C*	Characteristic value
100 ng/ml	13392	20244	0.661529362
10 ng/ml	2747	26454	0.103840627
1 ng/ml	442	35354	0.012502122
0.1 ng/ml	193	36069	0.005350855
0.05 ng/ml	318	39575	0.008035376
0	292	26233	0.011131018

**Table 3:** Linearity test.

Sample concentration	The value of *T*	The value of *C*	Characteristic value	Average value ($\bar{X}$)	Standard deviation (*σ*)	Coefficient of variation (CV)
100 ng/ml	13392	20244	0.661529362	0.698657612	0.032308661	0.046243913
	14896	20678	0.720379174			
	18430	25810	0.7140643			

10 ng/ml	2747	26454	0.103840627	0.115863316	0.015736094	0.135816017
	3928	29385	0.133673638			
	3131	28444	0.110075684			

1 ng/ml	442	35354	0.012502122	0.016030887	0.003334025	0.207975068
	595	36143	0.016462386			
	613	32047	0.019128155			

**Table 4:** Minimum test.

Sample concentration	100 ng/ml	10 ng/ml	1 ng/ml	0.1 ng/ml	0.05 ng/ml	0
Characteristic value	0.6987	0.1159	0.0160	0.0090	0.0085	0.0080

## Data Availability

The datasets generated and analyzed during the current study are not publicly available due to Tianjin University of Technology policy but are available from the corresponding author on reasonable request.
